# Relaxin gene family in teleosts: phylogeny, syntenic mapping, selective constraint, and expression analysis

**DOI:** 10.1186/1471-2148-9-293

**Published:** 2009-12-16

**Authors:** Sara V Good-Avila, Sergey Yegorov, Scott Harron, Jan Bogerd, Peter Glen, James Ozon, Brian C Wilson

**Affiliations:** 1 Department of Biology, University of Winnipeg, Winnipeg, Manitoba, R3E 2H9, Canada; 2 Department of Biology, Acadia University, Wolfville, Nova Scotia, B4P 2R6, Canada; 3 Department of Biology, Utrecht University, Utrecht, 3584 CH, Netherlands

## Abstract

**Background:**

In recent years, the relaxin family of signaling molecules has been shown to play diverse roles in mammalian physiology, but little is known about its diversity or physiology in teleosts, an infraclass of the bony fishes comprising ~ 50% of all extant vertebrates. In this paper, 32 relaxin family sequences were obtained by searching genomic and cDNA databases from eight teleost species; phylogenetic, molecular evolutionary, and syntenic data analyses were conducted to understand the relationship and differential patterns of evolution of relaxin family genes in teleosts compared with mammals. Additionally, real-time quantitative PCR was used to confirm and assess the tissues of expression of five relaxin family genes in *Danio rerio *and *in situ *hybridization used to assess the site-specific expression of the insulin 3-like gene in *D. rerio *testis.

**Results:**

Up to six relaxin family genes were identified in each teleost species. Comparative syntenic mapping revealed that fish possess two paralogous copies of human *RLN3*, which we call *rln3a *and *rln3b*, an orthologue of human *RLN2*, *rln*, two paralogous copies of human *INSL5*, *insl5a and insl5b*, and an orthologue of human *INSL3*, *insl3*. Molecular evolutionary analyses indicated that: *rln3a, rln3b *and *rln *are under strong evolutionary constraint, that *insl3 *has been subject to moderate rates of sequence evolution with two amino acids in *insl3/INSL3 *showing evidence of positively selection, and that *insl5b *exhibits a higher rate of sequence evolution than its paralogue *insl5a *suggesting that it may have been neo-functionalized after the teleost whole genome duplication. Quantitative PCR analyses in *D. rerio *indicated that *rln3a *and r*ln3b *are expressed in brain, *insl3 *is highly expressed in gonads, and that there was low expression of both *insl5 *genes in adult zebrafish. Finally, *in situ *hybridization of *insl3 *in *D. rerio *testes showed highly specific hybridization to interstitial Leydig cells.

**Conclusions:**

Contrary to previous studies, we find convincing evidence that teleosts contain orthologues of four relaxin family peptides. Overall our analyses suggest that in teleosts: 1) *rln3 *exhibits a similar evolution and expression pattern to mammalian *RLN3*, 2) *insl3 *has been subject to positive selection like its mammalian counterpart and shows similar tissue-specific expression in Leydig cells, 3) *insl5 *genes are highly represented and have a relatively high rate of sequence evolution in teleost genomes, but they exhibited only low levels of expression in adult zebrafish, 4) *rln *is evolving under very different selective constraints from mammalian *RLN*. The results presented here should facilitate the development of hypothesis-driven experimental work on the specific roles of relaxin family genes in teleosts.

## Background

The relaxin family of peptides belongs to the insulin superfamily and includes a group of signaling molecules that share similar gene and protein secondary structures. The genes have two exons that code for a prepropeptide consisting of a signal peptide, followed by B-, C-, and A-chains. Prohormone processing and activation occurs by removal of the C-chain by prohormone convertases that cleave at dibasic junctions [1]. In the mature peptide, six cysteine residues form three disulfide bonds that give this superfamily its distinctive secondary protein structure. In most mammals, the relaxin family consists of two relaxin peptides, RLN and RLN3, which share the receptor binding domain RXXXRXXI/V and four insulin-like peptides, INSL3, INSL4, INSL5, and INSL6, which have a less conserved motif [2]. Additionally, relaxin family peptides activate G protein-coupled receptors (GPCR) while other members of the insulin superfamily signal via tyrosine kinases [3].

The hormone RLN was the first member of the family to be studied in detail [2]. Originally characterized as a reproductive hormone [4], RLN is now implicated in diverse physiological processes, via its role in stimulating the production of matrix metalloproteinases (MMPs) which degrade extracellular matrix proteins and cause tissue remodeling [2]. By this action, the hormone is involved in parturition where it softens the connective tissues of the reproductive tract and prepares the mammary glands for lactation [2]; *RLN *has also been found to be involved in diverse processes involving tissue remodeling such as wound healing, angiogenesis and tumor formation [5,6]. In mammals, the *RLN *gene tandemly duplicated to give rise to two additional members of the family, *INSL4 *and *INSL6*, both of which are poorly understood, but which are both predominantly expressed in placenta and testis [7]. A more recent duplication of the *RLN *gene, specific to humans and anthropoid apes, resulted in primates having two copies of *RLN*, called *RLN1 *and *RLN2*, with *RLN2 *being functionally equivalent to the *RLN *in other mammals [2]. More recently, other members of the relaxin family have been identified: *RLN3 *was found to be expressed in the brain and testis of rodents and to exhibit high sequence conservation across mammalian species [8,9]. This led to predictions that RLN3 may function as a neuropeptide [8], which has received some empirical support because the peptide has been shown to be involved in the modulation of feeding activities, body weight regulation and in stress coordination, learning and memory [10,11].

Another member of the relaxin family, insulin-like peptide 3 (INSL3), attracted the attention of andrologists after it was discovered to play a crucial role in testicular descent in young males of human and mice [12]. There is also evidence that INSL3 is a survival factor for male and female germ cells in mammals [12,13]: it is expressed in significant amounts in testicular Leydig cells, while in females the distribution of INSL3 producing sites is less specifically localized, detected mainly in ovarian follicular theca cells [12]. However, the receptor for INSL3, RXFP2, has been identified in a broad range of tissues: brain, kidney, muscle, testis, thyroid, uterus, lymphocytes and bone marrow [12]. One of the least understood members of the relaxin family is INSL5, which was originally identified from analyses of expressed sequence tags in the human genome [14], and its expression has been detected in fetal brain, pituitary and colon as well as in the cortex of the thymus gland [15,16]. The receptor for INSL5, RXFP4, is broadly distributed in the human body, but the colon appears to be the most prominent site of RXFP4 mRNA expression. This is consistent with current hypotheses that it is involved in gut contractility and neuroendocrine signaling [15,16]. Thus, collectively, the relaxin family is revealing itself to be a group of peptides primarily involved in reproductive processes in mammals, and at the same time plays a broader role in other aspects of mammalian physiology.

Investigation on relaxin family peptides outside mammals has been limited. Relaxin-like peptides have been found in the testis, ovary, and/or alkaline glands of three species of sharks [17-21] and in bird testis [22]. However, there are only a few physiological studies on the expression of relaxin in teleosts [23,24], which are an infraclass of the bony fishes, and comprise 96% of the 26,000 ray-finned fish species and ~ half of all vertebrates on the planet [25].

Molecular estimates indicate that the common ancestor of teleosts and tetrapods existed ~ 450 million years ago (mya) [25,26]. A whole genome duplication (WGD), that occurred early in teleost evolution, ~ 350 mya, is hypothesized to have contributed to the rapid divergence of the group in part because of the opportunities that WGD's offer for acquisition of new gene functions [27,28]. After gene duplication, newly derived paralogous sequences are assumed to share similar functions to the ancestral gene. However, over time, the genes may be non-, sub- or neo-functionalized [29,30]. Because the teleost WGD event is ancient, examination of the proportional frequency and consequences of non-, sub- and neo-functionalization in teleosts have provided important insights into the role of gene duplication in vertebrates [31-35].

There have been two previous bioinformatics studies on the relaxin family [9,36]. Neither study focused on the molecular evolution nor expression of the family in teleosts, but they both included sequences of relaxin family genes from teleosts. Additionally, Park *et al*. [36] performed a syntenic data analysis of relaxin family genes in vertebrates and found that the common ancestor of teleosts and tetrapods harboured three independent relaxin family loci (*RFL*): *RFLA *- *INSL5*-like genes, *RFLB *- *RLN*-like genes and *RFLC *- *RLN3*-like genes. In this paper we expand upon these previous studies by including 32 relaxin family gene sequences from eight teleost species and by focusing our analyses on understanding the specific forces influencing orthologous and paralogous gene copy evolution of relaxins in teleosts. To this end, detailed analyses of teleost relaxin family genes were performed to assess the number of orthologous and paralogous sequences of relaxins in teleosts, their syntenic relationship to human relaxin family genes, the strength of purifying versus diversifying selection, the role of positive selection at the codon level, the relative expression of relaxin family genes in adult *Danio rerio *using real-time quantitative PCR (qPCR), and the site-specific expression of *insl3 *in *D. rerio *testis using *in situ *hybridization.

## Results

### Teleosts possess relaxin family sequences which are orthologous to four human relaxin genes

**Figure 1:**
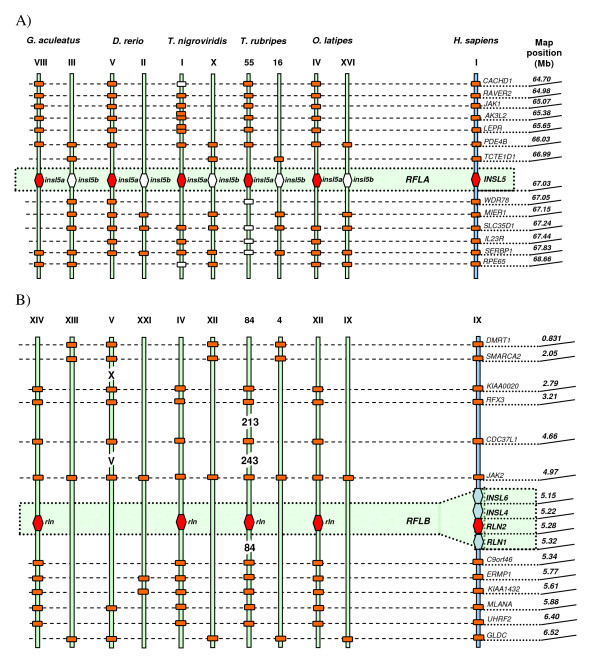
**Synteny maps**. Synteny maps comparing the orthologues of the relaxin family loci (*RFL*) and the genes flanking them in humans (*H. sapiens*) and five species of teleosts (*G. aculeatus, D. rerio, T. nigroviridis, T. rubripes *and *O. latipes*). 1A) *RFLA *locus contains the *INSL5 *gene in humans and its teleostean paralogues, *insl5a*/*insl5b; *1B) *RFLB *locus in humans hosts four relaxin family genes, namely *INSL6*, *INSL4*, *RLN2 *and *RLN1*; in teleosts this locus is represented by *rln *gene found as a single copy in all of the analyzed teleost genomes except for *D. rerio*, in which it is absent; 2A) *RFLCI *locus is represented by *RLN3 *in humans, and its paralogues, *rln3a*/*rln3b *in teleosts; 2B) *RFLCII *locus hosts *INSL3 *in humans, while 3 of the 5 studied teleosts contain single copy orthologues, *insl3*. The chromosome number (in Roman numerals) and map position of each gene in humans are given on the right. On the left, the genes orthologous to the human *RFL *are shown by orange hexagons in the central shaded section, and *RFL *paralogue that arose via the whole genome duplication shown as a white hexagon. Genes flanking the *RFL *that are syntenic in humans and teleosts are indicated by orange rectangles; the map position of each gene in teleosts is listed in Additional File 1: Table S3. Tandem duplicate copies of genes appear as two rectangles. Genes shown as white rectangles are genes identified on the same chromosome but in more distant locations (Additional File 1: Table S1). The genes *PDE4B/SLC35D1/SERBP1/RPE65 (RFLA); JAK2 (RFLB); TNPO2/RFX1/ASF1B/SLC27A1/GLT25D1 *(*RFLCI*); and *MED26/NR2F6/UNC13A/KCNN1/MAST3 *(*RFLCII*) were all retained in duplicate in 3 or more species (Additional File 1: Table S1). One gene, *NXNL1 RFLCI*) was retained tandemly duplicated in 3 species. Three of the 4 *RFL *linkage groups contained a copy of *JAK*, and 2 of the 4 contained copies of *PDE, SMARCA, RFX *and *MAST *genes.

**Figure 2:**
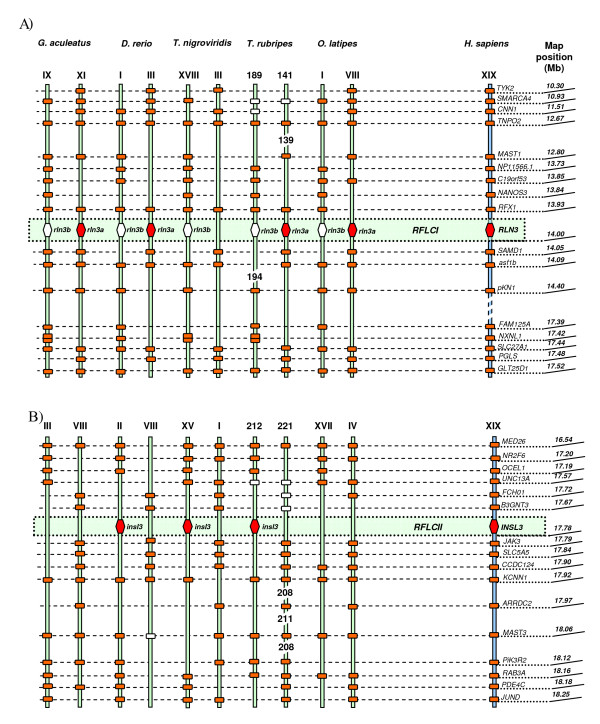
**Synteny maps**. Synteny maps comparing the orthologues of the relaxin family loci (*RFL*) and the genes flanking them in humans (*H. sapiens*) and five species of teleosts (*G. aculeatus, D. rerio, T. nigroviridis, T. rubripes *and *O. latipes*). 1A) *RFLA *locus contains the *INSL5 *gene in humans and its teleostean paralogues, *insl5a*/*insl5b; *1B) *RFLB *locus in humans hosts four relaxin family genes, namely *INSL6*, *INSL4*, *RLN2 *and *RLN1*; in teleosts this locus is represented by *rln *gene found as a single copy in all of the analyzed teleost genomes except for *D. rerio*, in which it is absent; 2A) *RFLCI *locus is represented by *RLN3 *in humans, and its paralogues, *rln3a*/*rln3b *in teleosts; 2B) *RFLCII *locus hosts *INSL3 *in humans, while 3 of the 5 studied teleosts contain single copy orthologues, *insl3*. The chromosome number (in Roman numerals) and map position of each gene in humans are given on the right. On the left, the genes orthologous to the human *RFL *are shown by orange hexagons in the central shaded section, and *RFL *paralogue that arose via the whole genome duplication shown as a white hexagon. Genes flanking the *RFL *that are syntenic in humans and teleosts are indicated by orange rectangles; the map position of each gene in teleosts is listed in Additional File 1: Table S3. Tandem duplicate copies of genes appear as two rectangles. Genes shown as white rectangles are genes identified on the same chromosome but in more distant locations (Additional File 1: Table S1). The genes *PDE4B/SLC35D1/SERBP1/RPE65 (RFLA); JAK2 (RFLB); TNPO2/RFX1/ASF1B/SLC27A1/GLT25D1 *(*RFLCI*); and *MED26/NR2F6/UNC13A/KCNN1/MAST3 *(*RFLCII*) were all retained in duplicate in 3 or more species (Additional File 1: Table S1). One gene, *NXNL1 RFLCI*) was retained tandemly duplicated in 3 species. Three of the 4 *RFL *linkage groups contained a copy of *JAK*, and 2 of the 4 contained copies of *PDE, SMARCA, RFX *and *MAST *genes.

**Figure 3:**
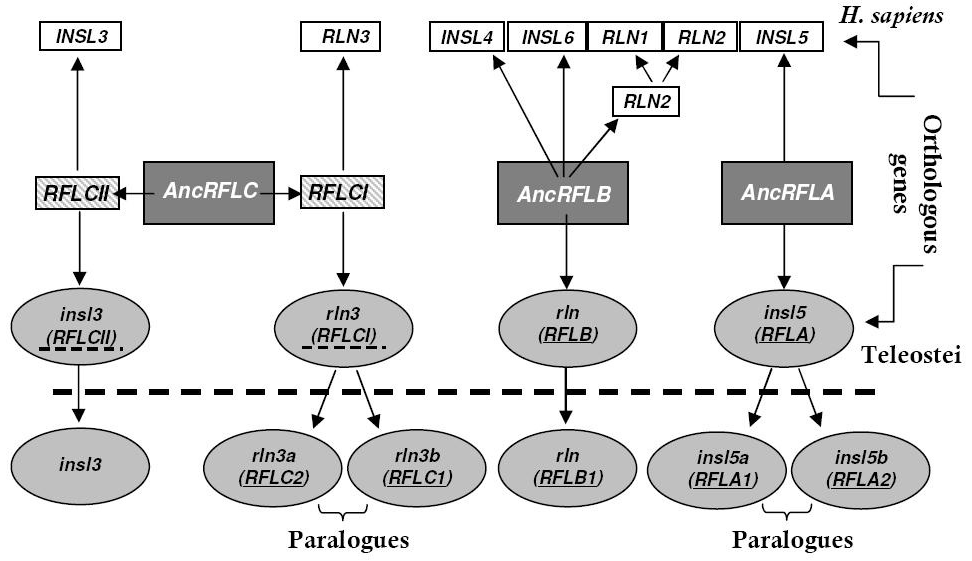
**Origins of relaxin family genes in teleosts (bottom) and humans (top) determined by synteny map analyses**. The ancestral Relaxin Family Loci (*AncRFL*) that are hypothesized to have been present in the common ancestor of teleosts and humans (tetrapods) are shown in the middle. We infer that AncRFLC duplicated giving rise to RFLCI (*rln3*) and RFLCII (*insl3*) prior to the divergence of teleosts and tetrapods. Names for the *RFL *proposed by Park *et al*. (2008) are given in brackets and underlined, those not used by Park *et al*., but inferred from this analyses are given in brackets with a dotted underline. The whole genome duplication (WGD) event resulted in two copies (paralogues) of each of the relaxin family genes in teleosts. *AncRFLA *gave rise to *INSL5 *and two paralogues in teleosts, *insl5a *and *insl5b*. *AncRFLB *was the predecessor of three human genes *INSL4, INSL6 *and *RLN2*, while the latter additionally underwent a recent duplication in primates producing *RLN1*. In teleosts, the *RFLB *gene, *rln*, is assumed to be orthologous to human *RLN2*. *AncRFLC *is hypothesized to have diverged into two loci: *RFLCI *harbouring *RLN3 *and the teleostean paralogues, *rln3a *and *rln3b*, and *RFLCII*, harbouring *INSL3 *and *insl3*. Duplicated copies of *insl3 *and *rln *in teleosts are believed to have been lost due to non-functionalization.

The syntenic data analysis showed that the six copies of relaxin family sequences analysed in the five teleost species are linked to four loci: two loci are syntenic to human *INSL5*, termed *RFLA *by Park *et al*. (2008), and harbour teleost *insl5a *and *insl5b *(Figure 1A); a locus syntenic to the human relaxin cluster (*RLN2/RLN1/INSL4/INSL6*) termed *RFLB *(Figure 1B), contains teleost *rln; *the locus syntenic to human *RLN3*, *RFLCI*, contains *rln3a *and *rln3b *(Figure 2A), and the locus syntenic to human *INSL3, RFLCII*, contains teleost *insl3 *(Figure 2B). *RLN3 *and *INSL3 *are ~ 3.8 Mb apart on human chromosome XIX, but they are not linked in teleosts; the genes linked to *rln *versus *insl3 *in teleosts split near the equivalent map position at 16 or 17 Mb of human chromosome XIX. A strong support was shown for the orthology between human and teleost relaxin family genes (Figure 1 and 2, Additional File 1: Table S1), although the linkage map for the human relaxin cluster containing *RLN1, RLN2, INSL4 *and *INSL6 *was less dense than that for the other chromosomes (Additional File 1: Table S1). Thus, of the six relaxin family genes in teleosts, four were present in the common ancestor of humans and teleosts and two arose as a result of the WGD in teleosts (Figure 3).

### Phylogenetic analysis reveals that all teleost relaxin family genes except relaxin group with their mammalian orthologues

**Figure 4:**
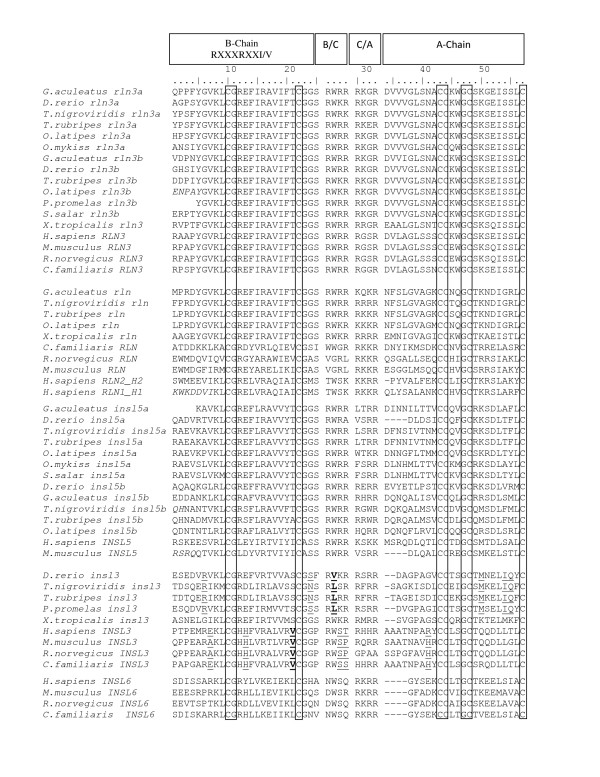
**Alignment of the deduced amino acid relaxin sequences from mammals and teleost species used for the phylogenetic analysis**. Conserved residues are boxed. Location of the relaxin receptor binding motif residues (RXXXRXXI/V), B-chain, A-chain, and twin dibasic junctions (B/C and C/A) are shown. Amino acids that are underlined are those identified as potential candidates of codon-specific positive selection using the branch-site model A analyses (see text for details), but only the two that are in bold and underlined were found to have a significant probability of being subject to positive selection with a BEB probability >0.95.

**Figure 5:**
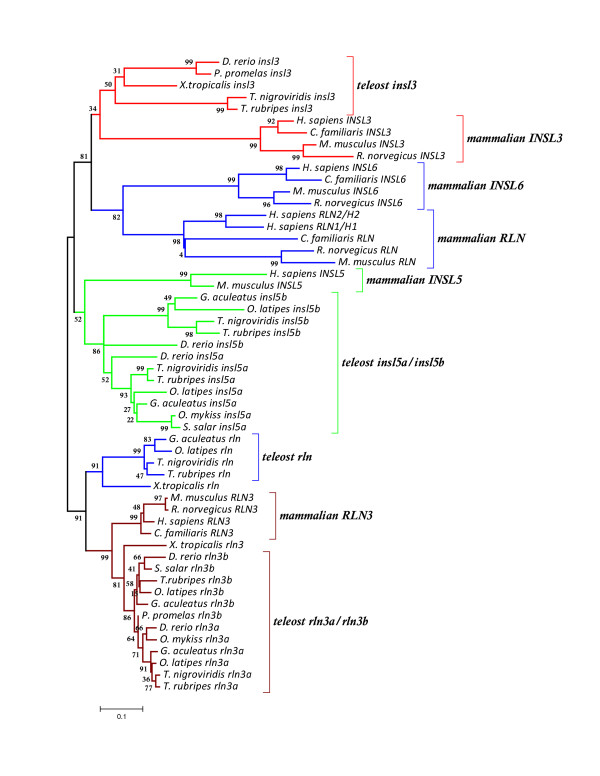
**Phylogenetic reconstruction of the relationship among relaxin family DNA sequences**. Phylogenetic tree reconstructed using the minimum evolution algorithm (a distance method) and including only the first two positions of each codon and employing the Tamura-3-parameter + Γ model of sequence evolution. Numbers at each node indicate the bootstrap values. Genes located at each of the four relaxin family loci, *insl5 (RFLA), rln (RFLB), rln3 (RFLCI) *and *insl3 (RFLCII)*, are shown in the same colour. Paralogous copies of *insl5 *(*insl5a *and *insl5b*) and *rln3 *(*rln3a *and *rln3b*) that arose after the teleost WGD are indicated. Mammalian *INSL6 *is a tandemly duplicated member of the relaxin family that is linked and paralogous to mammalian *RLN*.

Using the nucleotide alignment (Figure 4), hierarchical likelihood ratio tests indicated that the Tamura 3-parameter + Γ (with a = 1.24) model was the most appropriate model of DNA sequence evolution. This model was used for the minimum evolution tree based on the first two codon positions (Figure 5). For the Bayesian trees, the GTR + Γ model of sequence evolution was employed, and partitioning and unlinking the three codon positions revealed that the rate of change was approximately four times higher at the third compared to first and second codon positions (0.51, 0.40 and 2.00 respectively) and that the gamma parameter was 0.98 for the first two positions, but 5.06 for the third position. Hierarchical likelihood tests of the amino acid models included in ProTest v. 1.0.6 and Bayesian methods both strongly supported the WAG + Γ (a = 0.60) model of amino acid sequence evolution which was used to reconstruct the phylogenetic relationship among amino acid sequences (Additional File 2: Figure S1). Although the topology of the Bayesian partitioned DNA sequence tree was similar to the Bayesian tree based on amino acid data, the saturation of the third base position lowered confidence in the Bayesian Posterior Probabilities (BPP) and led to some problems of long-branch attraction: the two trees shown are the distance (minimum evolution) tree based on DNA (Figure 4) and the Bayesian topology based on the amino acid sequences using the WAG + Γ model of sequence evolution (Additional File 2: Figure S1).

The phylogenetic tree reconstructed from the DNA sequence data support the presence of four relaxin family groups in teleosts with reasonably high bootstrap support 1) *rln3a *and *rln3b *(81%) 2) *rln *(91%) 3) *insl5a *and *insl5b *(86%) and 4) *insl3 *(50%). All teleost relaxin family genes cluster with their mammalian orthologues as identified through the syntenic data analyses except teleost *rln*, which is sister clade to *rln3 *with high bootstrap support (91%) and exhibits, overall, a closer resemblance to these sequences (Figure 4) than to its true orthologue, mammalian *RLN*. The three frog sequences cluster with their orthologue basal to the teleost clade: in particular the *X. tropicalisrln *sequence shows greater similarity to teleost *rln *than to mammalian *RLN*. The BPP support for the Bayesian tree reconstructed with amino acid sequences gave similar results and statistical support (Additional File 2: Figure S1) to that based on DNA sequence data, with the main exception that teleost *insl3 *sequences have higher BPP support (74%) than based on the DNA sequence but they do not group with mammalian *INSL3*.

### Teleost relaxin family genes are subject to different levels of purifying selection

The two-cluster test was performed on the topology shown in Figure 5 and identified the following groups as having differential rates of evolution 1) teleost *rln3a *and *rln3b *exhibited accelerated evolution compared to mammalian *RLN3*, 2) teleost *insl5 *and *insl3 *independently exhibited accelerated evolution compared to teleost *rln3a*, *rln3b*, *rln *and mammalian *RLN3 *and 3) teleost *insl5b *showed accelerated evolution relative to *insl5a*.

The average value of d_n_/d_s _within each relaxin family gene or within the B- and A-chains ranged from 0.05 (*rln3b*, A-chain) to 0.48 (*insl5b*, A-chain) in teleosts and from 0.04 (*RLN3*, B-chain) to 0.78 (*INSL6*, B-chain) in mammals (Table 1). Values were generally lower in the B- compared to the A-chain. No gene was found to exhibit, overall, evidence of positive selection in which d_n_/d_s _> 1. In general, teleost *rln3a*, *rln3b *and *rln *had few non-synonymous changes in both the B- and A-chains; their d_n_/d_s _ratios were less than 0.10 indicating that they are under strong purifying selection comparable to mammalian *RLN3 *which had a d_n_/d_s _ratio of 0.08. Teleost *insl5a *exhibited similar and moderate levels of purifying selection to mammalian *INSL5*, with the d_n_/d_s _ratio in teleosts (0.25) being similar to that in mammals (0.23). On the other hand, teleost *insl5b *exhibited weaker purifying selection with an overall d_n_/d_s _ratio of 0.40, and having d_n_/d_s _ratios more than twice that for *insl5a *in the B- (0.34) and A-chains (0.48). Teleost *insl3 *exhibited similar (0.37) overall sequence divergence to *insl5b*. Although *insl5b *and *insl3 *are the teleost genes under the weakest evolutionary constraint (a result also supported by the molecular clock analyses), they are under stronger purifying selection than mammalian *RLN *and *INSL6*, which have d_n_/d_s _ratios of 0.64 and 0.46 respectively, with the B-chain of mammalian *INSL6 *exhibiting the highest rate of d_n_/d_s _at 0.78.

**Table 1:** Results of the analyses using the branch-site model A of Yang and Nielsen (2002) on relaxin family orthologues in teleosts.

Gene	Model	Foreground branch	Parameter	dF	2 Δ L	Positively selected sites
*insl5*	A (alt)	teleost *insl5b*	p0 = .55, p1 = .3, p2 = .14, ω_2 _= 1.0	3	0.0	--
	A (ω_2 = _1)	teleost *insl5b*	p0 = .53, p1 = .28, p2 = .17	2	--	N/A
	A (alt)	teleost *insl5a*	p0 = .52, p1 = .28, p2 = .2, ω_2 _= 1.04	3	1.0	--
	A (ω_2 = _1)	teleost *insl5a*	p0 = .51, p1 = .27, p2 = .22	2		N/A

*rln*	A (alt)	teleost *rln*	p0 = .43, p1 = .56, p2 = 0.0,ω_2_	3	0.0	--
	A (ω_2 = _1)		p0 = .43, p1 = .56, p2 = 0.0	2		N/A

*insl3*	A (alt)	teleost *insl3*	p0 = 0, p1 = 0, p2 = 1.0, 2^ = 1.0^	3	4.1	6R(.54), 24S(.83), 27V (.97),50M(.87), 54I (.91), 55Q(.64)
	A (ω_2 = _1)	teleost *insl3*	p0 = 0, p1 = 0, p2 = 1.0	2	-	N/A 7V(.75),13E(.87),21S
	A (alt)	mammalian *INSL3*	p0 = .56, p1 = .21, p2 = .23, ω_2 = _41.9	3	4.0	(.95),28K(.92),29R
						(.61),41G(.79)
	A (ω_2 = _1)	mammalian *INSL3*	p0 = .6, p1 = .22, p2 = .17	2		N/A

*rln3*	A (alt)	teleost *rln3*	p0 = 0, p2 = 1.0, ω_2 _= 999	3	0.48	All sites selected*
	A (ω_2 = _1)	teleost *rln3*	p0 = 0, p1 = 0, p2 = 1.0	2		--

For each gene, either teleosts or mammals were used as the foreground branch on which the alternate (alt) hypothesis of positive selection was compared to the null model (ω = 1, fixed). The proportion of sites subject to purifying (p0), nearly neutral (p1) and positive selection (p2) and the estimate of ω (ω_2_) in the free model are all given as is the Likelihood (L) of the model. The codon positions (using *D. rerio *as the reference sequence) of the sites estimated to be subject to positive selection are indicated where significant. The null and alternative models are significantly different when 2 Δ L > 3.841. *For rln3, the models were not statistically different.

The branch-site model A analyses indicated that only *insl3 *exhibited evidence of codon-specific selection within teleosts or mammals. The null model was rejected when both teleosts and mammals were used as the foreground branch and two amino acids were identified as being subject to positive selection with Bayes Empirical Bayes (BEB) values of > 0.95. Using teleosts as the foreground lineage, site 27 in the B/C cut site, which is valine/leucine in teleosts but tryptophan in mammals, shows evidence of positive selection. Setting mammalian *INSL3 *to the foreground branch, identified site 21 in the B-chain, which is fixed as valine in mammals but to serine in teleosts, is also under positive selection (Table 2). An additional 10 sites were identified by the branch-site model as being potential sites of selection but none had BEB values >0.95. However, it is interesting to note that of the 12 sites identified by the model, 3 are in the B/C cut site (Table 2, Figure 4).

**Table 2:** Proposed nomenclature for relaxin family loci (*RFL*) in teleosts.

Teleost gene name	Human	**Park *et al***.	**Wilkinson *et al***.	
			*T. rubripes*	*D. rerio*
*insl5a*	*INSL5*	*RFLA1*	*TrRLX3d*	*DrRLX3b*
*insl5b*	*--*	*RFLA2*	*TrRLX3e*	*DrRLX3d*
*rln*	*RLN*	*RFLB1*	*TrRLX3c*	*--*
*rln3a*	*RLN3*	*RFLC2*	*TrRLX3a*	*DrRLX3a*
*rln3b*	*--*	*RFLC1*	*TrRLX3b*	*--*
*insl3*	*INSL3*	RFLCII*	*TrRLX3f*	*DrRLX3c*

The proposed names for the six teleost *RFL *are given along with the nomenclature used for the orthologous genes in humans, and the nomenclature proposed by Park *et al*. (2008) and Wilkinson *et al*. (2005) in *Takifugu rubripes *and *Danio rerio *for teleost relaxin family genes. Park *et al*. (2008) propose that teleosts do not have an *insl3 *orthologue. They suggest that *insl3 *adopted its role in mammals after the divergence of Amphibia, and refer to the ancestral *insl3 *locus in amphibians as *RFLCII*.

### Quantitative PCR analysis showed evidence of significant expression of rln3a/rln3b and insl3 genes in the gonads and brain of zebrafish

**Figure 6:**
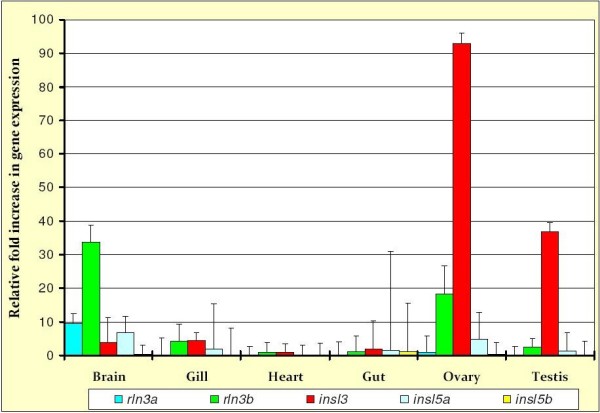
**Relative fold increase (with standard deviation) in mRNA expression of five relaxin family genes in six tissues relative to their expression in eye and normalized by the expression of the housekeeping gene, *b2m *(see text for details)**. Relative expression of relaxin family genes *rln3a*, *rln3b*, *insl3*, *insl5a *and *insl5b *were assessed in the brain, gill, heart, gut, ovary and testis dissected from adult zebrafish.

DNA sequencing of the products amplified using qPCR confirmed the identity of all *D. rerio *amplification products (not shown). The results of the expression analyses of relaxin family genes in *D. rerio *indicated that *rln3a *and *rln3b *are predominantly expressed in the brain, although *rln3b *was also expressed in the gonads, while *rln3a *was not (Figure 6). Additionally, the data strongly support a role for *insl3 *in both ovary and testes with additional expression of *insl3 *in the brain and gill: the expression of *insl3 *was not significantly lower than that of the housekeeping gene, *b2m*, in ovary, and was only marginally lower than *b2m *in testis (data not shown). Lastly, the results showed little evidence of expression of either *insl5a *or *insl5b *in any tissue: *insl5a *was expressed in most tissues (except heart) at low levels while *insl5b *showed uniformly low, essentially negligible, expression (Figure 6).

### In situ hybridization identified expression of insl3 in zebrafish testis in Leydig cells

**Figure 7:**
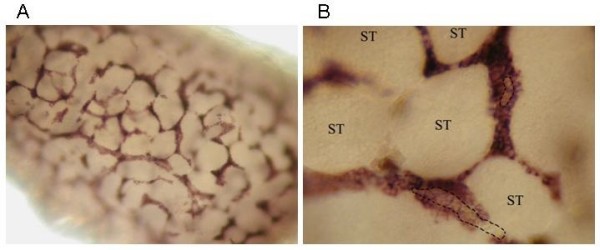
**Whole mount *in situ *hybridization of *insl3 *on zebrafish testis**. A) Overview of the positive *insl3 in situ *hybridization signal in zebrafish testis, clearly showing positive *insl3 in situ *hybridization signal in the interstitial area. B) Detailed view of A, showing that only the cytoplasm of the Leydig cells in zebrafish testis shows the positive *in situ *hybridization signal. Blood vessels (encircled by dashes) containing erythrocytes are often visible in the Leydig cell clusters. The seminiferous tubules (ST), containing Sertoli cells and germ cells in different stages of spermatogenesis, remain completely unstained.

A strong and specific signal of *insl3 *mRNA was observed in the interstitial area in the Leydig cells (Figure 7A). Higher magnification showed that the cytoplasm of these cells was strongly labeled while the nucleus remained unstained (Figure 7B). Positive cells formed clusters that were often arranged around blood vessels. There were no apparent (rostro-caudal or dorso-ventral) gradients in staining intensity in an adult testis, and all Leydig cells appeared to be labeled strongly. However, although not properly quantified yet, it is possible that the size of the Leydig cell clusters is larger in the periphery than in central areas of the testis. The intratubular area, containing Sertoli and germ cells at different stages of spermatogenesis, remained unlabeled. Taken together, these findings suggest that *insl3 *is a reliable Leydig cell marker in zebrafish testis tissue. No signal was observed with the sense cRNA *insl3 *probe (data not shown), indicating the specificity of the antisense probe generated against the sequence of zebrafish *insl3 *mRNA.

## Discussion

Reconstructing the evolutionary relationship among relaxin family genes in teleosts and tetrapods has highlighted the difficulties of determining orthologous and paralogous relationships in ancient gene families using phylogenetic data alone, in particular for small, relatively quickly evolving genes [25,26]. Previous phylogenetic studies on relaxin family genes [9,36] have also found a poor resolution of relaxin family genes, particularly in teleosts: by including more teleost species we find that only teleost *rln *failed to group with its mammalian orthologue and this is evidently due to very different selective pressures operating on the gene in the two groups (see discussion below). Using syntenic mapping data, we identify that the six relaxin family genes in teleosts are orthologous to four mammalian genes: *RLN3, RLN*, *INSL5 *and *INSL3 *with two of the genes, *INSL5 *and *RLN3*, containing paralogous copies in teleosts, *insl5a/insl5b *and *rln3a/rln3b *(Figure 3). These results are similar to those presented by Park *et al*. [36] with the exception that we find evidence that teleosts possess an orthologue to *INSL3*, while they argue that *INSL3 *arose via duplication from *RLN3 *after the divergence of teleosts from tetrapods. We therefore propose that the relaxin family genes that were first identified as *RLX3a-3f *by Wilkinson *et al*. [9] be named based on their orthology to the mammalian counterparts: *rln3a*/*rln3b*, *rln*, *insl5a*/*insl5b*, and *insl3 *respectively (Table 3). We encourage the adoption of this nomenclature, since there is currently considerable confusion regarding the identity of relaxin family peptides in teleosts on publically available databases.

**Table 3:** Average pairwise dn/ds values of relaxin family genes in teleost and mammals.

Relaxin family Gene	B + A - chain	B-chain	A-chain
	d_n_/d_s_ (d_s_, d_n_)	d_n_/d_s_ (d_s_, d_n_)	d_n_/d_s_ (d_s_, d_n_)
Teleost *rln3a*	0.08 (0.60, 0.05)	0.09 (0.57, 0.05)	0.07 (0.62, 0.04)
Teleost *rln3b*	0.07 (0.58, 0.04)	0.10 (0.57, 0.06)	0.05 (0.60, 0.03)
Teleost *rln*	0.09 (0.46, 0.04)	0.04 (0.45, 0.02)	0.11 (0.52, 0.06)
Teleost *insl5a*	0.25 (0.51, 0.13)	0.13 (0.53, 0.07)	0.20 (0.49, 0.10)
Teleost *insl5b*	0.40 (0.57, 0.23)	0.34 (0.58, 0.20)	0.48 (0.52, 0.25)
Teleost *insl3*	0.37 (0.63,0.23)	0.41 (0.64, 0.26)	0.45 (0.53, 0.24)
Mammalian *RLN*	0.64 (0.50, 0.32)	0.59 (0.56, 0.33)	0.60 (0.50, 0.30)
Mammalian *RLN3*	0.08 (0.51, 0.04)	0.04 (0.45, 0.02)	0.11 (0.56, 0.06)
Mammalian *INSL3*	0.36 (0.51, 0.18)	0.20 (0.50, 0.10)	0.40 (0.61, 0.24)
Mammalian *INSL5*	0.23 (0.79, 0.17)	0.19 (0.61, 0.12)	0.21 (0.98, 0.21)
Mammalian *INSL6*	0.46 (0.46, 0.20)	0.78 (0.32, 0.25)^+^	0.24 (0.58, 0.14)

Only the five teleost and four mammalian species for which complete genomic information was available were included in the analyses. Z-tests of whether d_n_/d_s _> 1 for any gene was performed using bootstrapping to compute the variance (+ p < 0.10, * p < 0.05). Average d_n_/d_s _< 0.1 represent strongly purifying selection, 0.11-0.5 represent weakly purifying seletion, and >1 represents recent positive selection. See text for details.

In teleosts, these relaxin family genes are subject to strong or moderate purifying selection: *rln3a, rln3b *and *rln *are all similar in sequence and highly conserved, *insl5a *exhibits a slightly faster rate of molecular evolution, and *insl3 *and *insl5b *exhibit the highest levels of molecular evolution in teleosts, the latter having a significantly faster rate of evolution than its paralogue *insl5a*. Using the branch-site model A test, we find evidence that one codon in teleost *insl3 *and another in mammalian *INSL3 *have been subject to positive selection. Lastly, we find evidence that five of the six relaxin family genes present in the model organism *Danio rerio *are expressed in one or multiple tissues, especially brain and gonads and that *insl3 *is specifically expressed in interstitial Leydig cells in zebrafish testis. The significance of these results will be discussed first within the context of the comparative analysis of orthologous relaxin family genes in teleosts and mammals and then with respect to the evolution and expression of paralogous relaxin family genes in teleosts.

### Relaxin family genes: teleosts versus mammals

#### Teleost rln is more similar in sequence to rln3 than to its mammalian orthologue RLN

Teleost *rln *was found in 4 of the 5 species for which the whole genome was available but it is, surprisingly, absent from the *D. rerio *genome. The close identity of teleost *rln *to *rln3*, and yet its striking difference from mammalian *RLN*, suggest that the gene has been subject to different evolutionary pressures in the two groups. Several processes could potentially have caused this such as: 1) teleost *rln *has retained the ancestral function of the gene while mammalian *RLN *has diversified in function or 2) teleost *rln *has undergone convergent evolution with *rln3*. Certainly there is support for the hypothesis that mammalian *RLN *has diversified in function: it exhibits the highest rate of molecular evolution of any of the relaxin family genes except *INSL6 *and it has duplicated giving rise to four fast-evolving paralogues in humans and anthropoid apes - *RLN1, RLN2*, *INSL4 *and *INSL6 *(Figure 1B), all of which are produced by human reproductive tissues [37,38]. Thus, the clustering of mammalian *RLN *with its linked paralogues, rather than with teleost *rln*, arises in part because the clade has a higher rate of evolution and more recent common ancestry than the clade harbouring teleost *rln*. This hypothesis is also supported by the phylogenetic clustering of frog and teleost r*ln *sequences (Figure 5). However, the identity of the B-domain of teleost *rln *with *rln3 *suggests that some factor such as shared receptor binding domains may have selected for them to retain the same sequence. Until the receptors of teleost relaxin family genes are known and the physiological role of teleost *rln *understood, it will be difficult to assess this hypothesis. Although the physiological role of relaxin in mammals is primarily associated with the reconstruction of connective tissue during reproduction [2], the gene is involved in several pathways not specific to reproduction, e.g. metalloproteinase activation, wound healing and reduction of fibrosis in non-reproductive tissues, that may reflect the ancestral role of the gene and its potential action in teleosts.

#### Teleost insl3 shows a similar spatial pattern of expression to mammalian INSL3

Since the descent of testicles from the abdominal cavity is solely specific to therian mammals, *insl3 *is postulated to have adopted this specialized role prior to the emergence of marsupials [36]. Park *et al*. [36] propose that the duplication of *RFLCI*, that gave rise to *RFLCII *harbouring *INSL3*, occurred prior to the divergence of amphibians and mammals but after the divergence of teleosts from tetrapods. They base this conclusion on the putative absence of an *INSL3*-like orthologue in fish. However, by studying more fish species, we find convincing syntenic evidence that the duplication of *RFLCI *and *RFLCII *occurred prior to the divergence of fish and tetrapods (Figure 3). Indeed, our qPCR results show that *insl3 *was the most abundantly expressed relaxin in *D. rerio *and that it was highly expressed in both ovary and testis, exhibiting only marginally lower expression levels than the housekeeping gene. The *in situ *hybridization results additionally showed that *insl3 *is expressed in the interstitial area of *D. rerio *testis (i.e. in Leydig cells) but is completely absent from the intratubular section (containing Sertoli and germ cells). This pattern of Leydig cell-specific staining has also been identified for Cyp17a1 [39] and for 3βHSD [40], both Leydig cell proteins involved in zebrafish germ cell sexual differentiation.

Park *et al*. [36] show how specific changes to the receptors for mammalian RLN3 and INSL3, RXFP1 and RXFP2 respectively, during early therian evolution allowed for INSL3 to adopt its specific role in testicular descent in mammals: they further show that the gene products of *RFLCI *(*rln3a *and *rln3b*) activate both rxfp1 and rxfp2 in teleosts. A role for codon-specific positive selection in the evolution of the *insl3 *gene was also found in this study: *insl3 *was the only relaxin family gene which exhibited evidence of lineage and site-specific selection in teleosts and mammals. Two amino acids were found to show evidence of positive selection at the 95% significance level when teleosts or mammals were used as the foreground lineage, although a total of twelve sites were included in the most probable posterior model. Interestingly, three of these twelve sites were in the B/C dibasic junction. Prohormone convertases activate hormones by cleaving dibasic chain junctions; our results suggest that different expression patterns between mammalian and teleost relaxin family genes may be mirrored by these convertases [41,42]. Overall, our data support Park *et al*.'s [36] conclusion that the co-evolution of INSL3-RXFP2 may have allowed INSL3 to adopt its particular role in mammalian testicular descent, but we also show that *INSL3-*like genes are present in teleosts and that they are also involved in Leydig cell differentiation.

#### Teleost rln3 paralogues show similar gene sequence and expression to mammalian RLN3

Examining the spatial and temporal expression of *rln3 *paralogues, Donizetti *et al*. [23] recently found evidence for the expression of both genes in adult zebrafish brain. Additionally, they found expression during larval stages for *rln3a *in the nucleus incertus and for *rln3b *in the periaqueductal gray (PAG) matter, the latter being implicated in vocal communication in fish. Our qPCR results in adult zebrafish are consistent with theirs, and the putative role of RLN3 as a neuropeptide involved in feeding, body weight regulation, stress coordination, learning and memory [10,11]. Interestingly, while we find significant expression of *rln3b *in the ovary, Donizetti *et al*. [23] showed evidence of expression of *rln3b *in the testis but not the ovary of adult zebrafish. Even though the difference between the expression of *rln3b *in the two sexes deserves further attention, the hypothesis that *rln3 *performs a dual function in teleosts is supported by the work of McGowan *et al*. [43]. They found evidence for the involvement of *RLN3 *in the hypothalamic-pituitary-gonadal axis in mice, suggesting that it may be a signal linking nutritional status and reproductive function. Collectively, these data suggest that *rln3 (RLN3) *may play similar roles in teleosts and mammals, which is further supported by its high degree of sequence conservation between the two groups [[9], this study].

#### Both teleost insl5 paralogues are well represented but, as in mammals, their role(s) are unclear

Seven of the eight examined species of teleosts harboured *insl5a *genes, and all the species for which the whole genome had been sequenced, additionally contained the paralogous sequence *insl5b*. Despite its presence in the genome, the qPCR data in *D. rerio *were more ambivalent. While some expression of *insl5a *was found in several tissues, particularly brain and gill, only very low expression of *insl5b *was found in the examined adult zebrafish tissues.

#### Evolution and expression of paralogous genes, rln3 and insl5, in teleosts

Our data show that *rln3a/3b *and *insl5a/5b *arose by duplication after the tetrapod-teleost divergence. It has been proposed that duplicate gene copies may 1) accumulate nonsense mutations in regulatory or gene elements and become non-functionalized, 2) diverge in the tissue or timing of expression compared to the ancestral copy and become sub-functionalized, or 3) acquire new functions and be neo-functionalized [30]. Theory suggests that duplicated genes are most likely to be lost or sub-functionalized [44], and genome wide scans indicate that about 80%-85% of teleost genes were non-functionalized after the WGD [29,45]. The relative rates of sub- versus neo-functionalization are difficult to determine, in part because sub-functionalization may lead to neo-functionalization over long evolutionary timescales [46]. Additionally, changes associated with sub-functionalization often occur in promoter regions that regulate timing or control of gene expression and studies that examine the rate or pattern of molecular evolution in the protein coding region alone may not detect sub-functionalization [44].

The data presented here suggest that the paralogous copies of *rln3 *and *insl5 *may have been subjected to different forces of "functionalization" post-duplication. The paralogues *rln3a *and *rln3b*, exhibit similar patterns of molecular evolution consistent with sub-functionalization of the duplicated copy. Our qPCR data indicate that *rln3b *is expressed in the brain and ovary while *rln3a *is expressed only in the adult zebrafish brain. This result is consistent with the findings of Donizetti *et al*. [23] although they additionally find evidence of distinct differential expression of these two paralogues during zebrafish embryogenesis. Seemingly, the pattern observed in adult zebrafish for *rln3 *paralogues differs somewhat from the classical definition of sub-functionalization because sub-functionalized copies are expected to diverge in temporal or spatial expression but collectively span the ancestral expression patterns, although our results are consistent with other data on the expression of paralogous genes [45,46]. On the other hand, the duplicated copies of *insl5 *appear to be subject to different selective pressures. The molecular clock analyses revealed that *insl5b *has had an accelerated rate of evolution compared to *insl5a*, and the average value of d_n_/d_s _was more than twice as high in the B- and A-chains of *insl5b *compared to *insl5a*, a pattern identified for other duplicated teleost genes believed to have undergone neo-functionalization [45]. Support for the contention that *insl5a*, rather than *insl5b*, has retained the ancestral function is further given by the syntenic data analyses in which many of the genes linked to *INSL5 *in humans are preferentially linked to *insl5a *(Figure 1A). The low levels of expression for *insl5a *and even lower levels for *insl5b *suggest that either 1) we have not identified the main tissues of expression for these genes and/or 2) they are expressed at developmental stages not included in this preliminary analysis (adult male and female zebrafish): in the future this will be explored using more detailed qPCR studies.

## Conclusions

We find that teleosts harbour orthologues of four mammalian relaxin family genes: *RLN, RLN3, INSL3 *and *INSL5*. Two of the orthologues exist as paralogous duplicates in teleosts (*rln3a/rln3b *and *insl5a/insl5b*) probably as a result of the WGD event that occurred early in the evolution of teleosts. By combining the bioinformatics and expression analyses performed in this study we can draw the following conclusions about each teleost relaxin gene: 1) both *rln3 *paralogues exhibit similar evolution and expression to mammalian *RLN3 *and the paralogous copies appear to have been sub-functionalized, 2) teleost *insl3 *has evolved moderately quickly like its mammalian counterpart and shows similar tissue-specific expression in Leydig cells, has undergone site-specific codon selection in both teleosts and mammals, and additionally exhibited high expression in the ovary of teleosts, 3) *insl5 *genes are well represented in teleosts, *insl5a *exhibits similar rates of evolution to *insl3*, while *insl5b *shows accelerated evolution compared to *insl5a *and may have been neo-functionalized, 4) molecular evolutionary analyses indicate that teleost *rln *is operating under very different selective constraints from mammalian *RLN*, and appears to mimic *rln3 *in its sequence evolution. Taken together, these results underscore the diverse roles that relaxin family peptides must play in teleosts: further experimental work is needed to shed light on the similarities and differences of their physiological roles in teleosts.

## Methods

### Nomenclature of teleost relaxin family genes

Wilkinson *et al*. (2005) identified six copies of relaxin in *Takifugu rubripes *and called them *RLX3a *through *RLX3f*. Recently, using both syntenic and phylogenetic data, Park *et al*. (2008) estimated that the ancestor of tetrapods and teleosts harboured three relaxin family loci (*RFL*): *RFLA *- hosting *INSL5*-like genes, *RFLB *- containing *RLN*-like genes and *RFLCI *- including *RLN3*-like genes; they suggest that the duplication of *RFLCII *that gave rise to *INSL3 *occurred just prior to or after the divergence of Amphibia. These previous studies included 11 [9] and 14 [36] teleost sequences, and focused on resolving the phylogenetic and molecular evolutionary patterns of the relaxin family in tetrapods. Here, by searching the genomic databases of five completed teleost genomes, and including 32 teleost sequences, our results generally support the conclusions of Park *et al*. [36], except that we find that teleosts harbour an orthologue to human *INSL3*, indicating that the duplication of *RFLC *occurred prior to the divergence of teleosts and tetrapods. The phylogenetic and syntenic data analyses presented below indicate that the genes originally called *RLX3a*-*3f *by Wilkinson *et al*. [9] pertain to four relaxin family loci and are more accurately named *rln3a, rln3b, rln, insl5a, insl5b*, and *insl3 *respectively. The orthologous relationship of these genes to human relaxin family genes and their equivalence in the nomenclature of Wilkinson *et al*. [9] and Park *et al*. [36] is provided (Table 3).

### Sequence identification and syntenic relationship of relaxin family genes in teleosts

Publicly available databases were searched for relaxin family homologues in the five teleost species for which a significant region of the genome has been sequenced: *Tetraodon nigroviridis *version 7 (Jaillon *et al*., 2004, http://www.ensembl.org/Tetraodon_nigroviridis/Info/Index ), *Takifugu rubripes *version 4 (Aparicio *et al*. 2002, http://www.ensembl.org/Takifugu_rubripes/Info/Index ), *Danio rerio *version 6 (The Wellcome Trust Sanger Institute, http://www.sanger.ac.uk/Projects/D_rerio and as available at Ensembl, http://www.ensembl.org), *Oryzias latipes *version 1 (Medaka Genome Project, http://dolphin.lab.nig.ac.jp/medaka) and *Gasterosteus aculeatus *version 1 http://www.ensembl.org/Gasterosteus_aculeatus/Info/Index . The sequences were first identified by using the algorithms BLASTP and TBLASTN to search for the following *D. rerio *B-chain protein sequences: YGVKLCGREFIRAVIFTCGGSRW (*rln3b*), RTVKLCGREFIRAVVYTCGGSRW (*insl5a*), and VRVKLCGREFVRTVVASCGSFRV (*insl3*). High-scoring hits (> 65% sequence identity over the entire region) were identified and then the upstream and downstream regions of the candidate relaxin family genes were searched for complete open-reading frames and other relaxin motifs, such as the conserved A-chain structural motif (CXXXCX_8_C), the B/C and C/A dibasic junctions, and the B-chain relaxin receptor binding motif RXXXRXXI/V, as well as general gene structure before being included in the data analyses. In total 26 genes were identified from these five genomes, 23 of the genes are annotated and identified as belonging to the *RLN/INSL *family in Ensembl (release 54); all of the relaxin family genes exhibited the expected gene structure for the family, complete open reading frames even through the post-translationally cleaved C-peptide. The Ensembl gene ID of these 23 genes as well as the location of all 26 genes is given (Release 54, Appendix Table two).

All known mammalian relaxin sequences were obtained from *Homo sapiens RLN1, RLN2*, *RLN3*, *INSL3, INSL 5 *and *INSL 6*; *Mus musculus RLN*, *RLN3, INSL3, INSL5 *and *INSL6*; *Rattus norvegicus RLN, RLN3, INSL3 *and *INSL6; *and *Canis familiaris RLN, RLN3, INSL3 *and *INSL6 *from GenBank http://www.ncbi.nlm.nih.gov/ , and all known relaxin sequences from *Xenopus tropicalis *were included. Additionally, six relaxin family genes were identified from other teleost species from cDNA or EST databases at NCBI: *Oncorhynchus mykiss rln3a *and *insl5b*; *Pimephales promelas rln3b *and *insl3*; and *Salmo salar rln3a *and *insl5a *(Appendix Table two) such that a total of 32 teleost relaxin family genes were included. Mammalian sequences for *INSL4 *were not included in the analysis because the gene contains a large insertion, is present only in mammals, and was the subject of a previous bioinformatic analysis [9].

A syntenic analysis of the relationship between teleost and mammalian relaxin family genes was performed by identifying the position of up to ten genes both up- and downstream of the focal genes in *D. rerio, T. nigroviridis*, *T. rubripes*, *O. latipes *and *G. aculeatus*. Syntenic maps were constructed based on the information regarding the location of genes available from Ensembl's BioMart data mining tool http://www.ensembl.org/multi/martview and, as needed, verified using the UCSC Genome Bioinformatics web server http://genome.ucsc.edu.

### Phylogenetic analyses

For all sequences, the location of the signal peptide was determined using SignalP 3.0 [47] using default settings and then the sequence was removed. Sequence alignment was accomplished by manually aligning the translated B- and A-chain conserved motifs and twin dibasic motifs, the latter correspond to the protease cleavage sites between the B/C and C/A chain (Figure 4). The sequence between the two twin dibasic motifs was removed before alignment, because it contained the non-conserved intron and C-chain, but the entire B- and A-domains plus the four amino acids at the B/C and C/A protease cleavage sites were included since the latter could be potential targets of selection (Figure 4). Relaxin family members have classically been distinguished by the presence of the receptor binding RXXXRXXI/V motif on the B-chain; however, this is not specific enough to identify individual teleost relaxin family genes. Therefore, sequence motifs for the B-chain and dibasic cut sites were identified to characterize potentially important structural and functional residues and to aid in distinguishing teleost relaxin family genes (Additional File 1: Table S3). Teleost *rln3a*, *rln3b *and *rln *share an identical strongly conserved B-chain motif that is also shared by mammalian *RLN3 *but teleost *rln *differs from teleost and mammalian *RLN3 *in its C/A dibasic motif. Teleost *insl5a *and *insl5b *are less conserved than *rln3a*, *rln3b *and *rln *and contain unique but related B-chain and dibasic motifs. Finally, *insl3 *contains the least conserved B-chain but has specific dibasic processing sites that distinguish it from the remaining relaxin family genes (Additional File 1: Table S3).

The most appropriate model of nucleotide sequence evolution was identified using likelihood ratio tests as implemented in Model Test [48]. The phylogenetic relationship among nucleotide sequences was reconstructed using the optimality criteria of both minimum evolution and Bayesian methods as implemented in MEGA 3.1 [49] and MrBayes 3.12 [50] respectively. Preliminary analyses indicated that variation at the third position was saturated and confounded resolution at deep internal nodes. Therefore, trees based on nucleotide data were reconstructed in MrBayes by partitioning the data into first, second and third codon position, and allowing each partition to evolve at its own rate with its own shape (gamma) parameter, or by including only the first two positions when minimum evolution was the optimality criteria. The relationship among amino acid sequences was also reconstructed using Bayesian methods available in MrBayes 3.12 [50] and the appropriate model of amino acid change was determined using hierarchical likelihood tests as implemented in ProTest version 1.0.6 [51] or by Bayesian methods. For the Bayesian analyses, the model of amino acid change was examined by allowing the parameter space explored by the MCMC algorithm in MrBayes to include eight different amino acid models (prset = mixed) and then choosing the model with the highest posterior probability as the best available model. For the Bayesian analyses of both the amino acid and nucleotide data, the MCMC algorithm was run with four simultaneous chains that sampled from the posterior distribution every 300 generations; trees sampled before the cold chain reached stationarity based on plots of the maximum likelihood scores were discarded as "burnin" while sampling continued until convergence was achieved based on the average standard deviation of the split frequencies and the potential scale reduction factor (PSRF) as given in MrBayes. Statistical confidence in the deduced evolutionary trees was assessed by examining the Bayesian Posterior Probabilities (BPP) on the majority-rule consensus tree containing branch lengths for the Bayesian analyses or by bootstrapping the sequences for 1000 generations for the minimum evolution analyses.

### Evidence for selection at the gene and codon level

To test whether the rates of molecular evolution were homogeneous across gene families, the two-cluster test was employed [52]. This test identifies those clades/genes that have significantly different rates of substitution based on an *a priori *hypothesis about which clades should be examined. Here, the rate of evolution was compared in nine clades: teleost *rln3a, rln3b, rln, insl5a, insl5b *and *insl3 *and mammalian *RLN3, INSL3 *and *INSL5*, while mammalian *RLN *and *INSL6 *were used as outgroups. The two-cluster test was conducted on the phylogenetic tree generated using minimum evolution using the program TPCV in LINTREE and only those comparisons with Z-scores high enough to give a p < 0.01 were taken as significant.

To assess the strength of purifying selection among genes, we calculated the average proportion of mutations leading to synonymous (d_s_) versus non-synonymous (d_n_) changes for all orthologous relaxin family sequences separately in teleosts and mammals. The ratio of d_n_/d_s _was calculated in MEGA 3.1 using a Nei-Gojobori model of nucleotide substitution [49]. Pairwise comparisons between teleost relaxin family genes were performed and average d_n_/d_s _values calculated across the entire gene, or for only the B-chain and A-chains; additionally the codon-based Z-test was used to determine if d_n_/d_s _within each gene or gene region was significantly different from 1.0 using bootstrapping to estimate the variances [49]. Because d_n_/d_s _values are calculated pairwise and the average value from all pairwise comparisons reported, the same five teleost species (those for which the whole genomes were available) and four mammalian sequences were included in these analyses.

Positive selection is often restricted to specific lineages and a few amino acid sites, therefore, we further employed the branch-site model A [53] to look for evidence of codon-specific positive selection on orthologous gene families in teleosts versus mammals. The application of this model requires that the user specify *a priori *which branch is being tested for evidence of positive selection, the so-called foreground branch, while the remaining groups are defined as background branches. Tests of positive selection were made by comparing the branch-site model A in which ω (d_n_/d_s_) ≥ 1 (alternative hypothesis) to the model A in which ω = 1 fixed (null hypothesis) and setting the foreground branch to the base of the clade containing the relaxin family orthologue in teleosts and the background branch was set to the same orthologue in mammals or vice versa [54]. Analysis of the branch-site model A was done using CODEML from the PAML package (PAML v. 4.2); models were compared using the Likelihood Ratio Test with 1 degree of freedom and, where significant, the posterior probability that a codon was under positive selection was estimated using the Bayes empirical Bayes (BEB) procedure [55].

### Expression of relaxin family genes in zebrafish using real-time, quantitative PCR

We tested for the expression of *rln3a*, *rln3b, insl5a, insl5b *and *insl3 *in *D. rerio*, which lacks *rln *(Appendix, Table two), using real-time, quantitative PCR (qPCR). Total RNA was extracted from the brain, heart, gill, gut, ovary, testis, and eye of adult zebrafish using the Aurum total RNA mini kit (BioRad) and first strand cDNA synthesized from 5 μg of total RNA with oligo dT and random hexamer priming (iScript Select cDNA Synthesis Kit, BioRad).

The relative transcript abundance of the five relaxin family genes in *D. rerio *across tissues was then calculated via qPCR using a MiniOpticon Real-Time detection system (BioRad). Oligonucleotide primers for *rln3a, insl5a *and *insl3 *were taken from Wilson *et al*. (2008), while those for *rln3b *and *insl5b *were designed using PRIMER3 software [56]. The primers selected for *rln3b *were: F:5'-CGGCTCTCGTAGTGTGTCTG-3'and R:5'-CCTGTTCACCTTGTCCCAGT-3' and for *insl5b *were: F: 5'GCTCAGGCACAGAAAGGTCT-3' and R: 5'-GCTGGAGTCCTGTGCTTCTC-3'. The iQ™SYBR^® ^Green Supermix kit was used according to the manufacturer's suggested protocols (BioRad). Standard curves were generated for all of the used primers to compute the amplification efficiency values for each primer set. The insignificant difference observed among the calculated efficiency values permitted us to calibrate Ct values of the target relaxin family genes in each tissue relative to their expression in eye (low abundance transcript, used as the calibrator in the equation below), and normalize them to a reference, housekeeping gene (*b2m*), previously shown to exhibit consistent expression across sexes, tissues and developmental stages in *D. rerio *[57]. This further allowed us to determine the relative fold increase of each relaxin gene relative to the housekeeping gene according to the formula: [58]. Each gene was tested three times and standard errors were calculated so that comparisons could be made across genes and tissues using the coefficient of variation (CV) where: .

### In situ hybridization using insl3 on zebrafish testis

A zebrafish-specific *insl3*-specific PCR product was generated with primer 2126 (5'- GGGCGGGTG**TTATTAACCCTCACTAAA**GGGAGTGAAGATGTGCGAGTGAAGC-3'; containing the T3 RNA polymerase promoter sequence [underlined]) and primer 2127 (5'-CCGGGGGGTG**TAATACGACTCACTATA**GGGGTACTGAATCAGTT CATTCATGGTGCA-3'; containing the T7 RNA polymerase promoter sequence [underlined]). The ~ 450 bp PCR product was gel purified, and served as a template for digoxigenin-labeled cRNA probe synthesis. For digoxigenin-RNA labeling by *in vitro *transcription, 500 ng PCR product was incubated at 37°C for 2.5 h in a 20 μl reaction volume containing 4 μl 5 × T3/T7 RNA buffer (Invitrogen), 2 μl 0.1 M DTT, 1 μl (29.7 units/μl) RNAguard (GE Healthcare, Fairfield, CT, USA) and 2 μl 10 × DIG RNA labeling mix (Roche), and either 2 μl (50 units/μl) T3 RNA polymerase (Epicentre; for sense cRNA probe synthesis) or 2 μl (50 units/μl) T7 RNA polymerase (Epicentre; for antisense cRNA probe synthesis).

To visualize cellular expression sites of *insl3 *mRNA in zebrafish testis, whole mount *in situ *hybridization was performed on zebrafish testicular tissue, fixed in 4% paraformaldehyde in PBS (pH 7.4), as described by Westerfield (2000) http://zfin.org/zf_info/zfbook/chapt9/9.82.html with some modifications to the protocol. Briefly, tissue was treated with 20 μg/ml proteinase K (Sigma-Aldrich) at 37°C for 20 min. Moreover, after post-fixation and before pre-hybridization, an acetic anhydride (0.25% in 0.1 M triethanolamine; Merck) treatment was included to reduce background. After termination of NBT/BCIP (Sigma-Aldrich) staining with 3 consecutive PBS washings, tissue was examined with a binocular connected to a digital camera.

## Authors' contributions

SVG-A. Oversaw the project, performed the phylogenetic, d_n_/d_s_, molecular clock, synteny, and codon selection analyses and wrote the bulk of the final paper. SY performed the qPCR laboratory work and analyses, helped with the synteny analyses, drew the synteny figures and helped with the literature search and writing and editing of the manuscript. SH helped collect the sequences, trimmed and aligned them, and devised the teleost gene motifs and helped write a previous version of the paper. JB performed the *in situ *hybridization analyses and helped with revising the manuscript, PG and JO both helped collect the original sequences and JO tabulated most of the information in Appendix 1, BW helped edit the manuscript and initiated the study. All authors have read and agreed to the final version of the manuscript.

## Supplementary Material

Additional file 1 **Tables describing the map positions of genes covered in the synteny analyses, sequence accession numbers for all relaxin family genes included in the paper and additionally the map positions of those in teleosts, and finally B-chain and dibasic junction sequence motifs of teleost relaxin family genes.** Table S1 covers the chromosomal map position of all genes used in the synteny analyses in each of the five teleost genomes on which syntenic mapping was conducted. Table S2 covers the NCBI or Ensemble accession number for each of the relaxin family loci presented, and, for the teleost genes, additionally the map position, intron and C-domain length. Table S3 presents the conserved B-chain motifs and B/C and C/A dibasic junctions for each of the teleost relaxin family loci. Click here for file

Additional file 2 **Bayesian phylogenetic tree based on relaxin family protein sequences. **The figure legend and Bayesian phylogenetic tree (in colour) based on amino acid sequences of relaxin family sequences from teleosts and mammals in pdf format. Click here for file
