# Supplementary material for: Relaxin gene family in teleosts: phylogeny, syntenic mapping, selective constraint, and expression analysis
Source: BMC Evol Biol. 2009 Dec 16;9:293. doi: 10.1186/1471-2148-9-293 (PMC2805637; doi:10.1186/1471-2148-9-293)
Supplement: Additional file 1 — Tables describing the map positions of genes covered in the synteny analyses, sequence accession numbers for all relaxin family genes included in the paper and additionally the map positions of those in teleosts, and finally B-chain and dibasic junction sequence motifs of teleost relaxin family genes. Table S1 covers the chromosomal map position of all genes used in the synteny analyses in each of the five teleost genomes on which syntenic mapping was conducted. Table S2 covers the NCBI or Ensemble accession number for each of the relaxin family loci presented, and, for the teleost genes, additionally the map position, intron and C-domain length. Table S3 presents the conserved B-chain motifs and B/C and C/A dibasic junctions for each of the teleost relaxin family loci. [file 1471-2148-9-293-S1.DOC]

Table S1. Map position of genes flanking Relaxin Family Linkage (*RFL*) groups in teleosts identified via syntenic mapping of the same genes in humans (see text). Map positions taken from those identified at ensemble.org. Some genes were unplaced (UN), or did not appear to exist (--), others were located on unassembled scaffold (Sc+number), and a few could not be positively identified (?).

**Linkage group name:** *RFLA-insl5*

**Location in human genome**: Chromosome I

| **Gene name** | **Map position in teleosts (MB)**  **(Chromosome numbers given in roman numerals, map positions presented with arabic numerals )** | | | | | | | | | |
| --- | --- | --- | --- | --- | --- | --- | --- | --- | --- | --- |
|  | *G. aculeatus* | | *D. rerio* | | *T. nigroviridis* | | *T. rubripes* | | *O. latipes* | |
|  | VIII | III | VI | II | I | XV | 55 | 166 | IV | XVII |
| *CACHD1* | 7.8 |  | 16.8 |  | Unplaced | | 1.14 |  | 16.8 |  |
| *RAVER2* | 7.9 |  | 16.7 |  | 15.7 |  | 1.17 |  | 16.8 |  |
| *JAK1* | 7.5 |  | 16.5 |  | 15.7 |  | 1.1 |  | 16.7 |  |
| *AK3L2* | 8.0 |  | 16.6 |  | 15.7 |  | 1.23 |  | 16.7 |  |
| *LEPR* | 8.04 |  | 16.4 |  | 15.8 |  | 1.27 |  | 16.6 |  |
| *PDE4B* | 8.1 | 9.8 | 16.1 |  | 15.5 | 2.9 | 1.3 | UN | 16.3 | 16.3 |
| *TCTE1D1* |  | 9.5 | -- | -- |  | 3.4 |  | .54 | ? |  |
| ***insl5*** | 8.1 | 9.5 | 15.9 | 6.2 | 15.6 | 3.4 | 1.3 | .54 | 16.5 | 16.7 |
| *WDR78* |  | 4.2 | 15.9 |  | ? |  | Sc74 |  | -- |  |
| *MIER1* |  | 9.5 | 51.1 | 2.5 |  | 3.4 |  | .54 |  | 16.8 |
| *SLC35D1* |  | 9.5 | 51.1 |  | 2.9 | 3.4 | Sc13 | .53 | 3.4 | 16.8 |
| *IL23R* | Unplaced | | 51.2 |  | 3.0 |  | Sc25 |  | 28.1 |  |
| *SERBP1* | 19.05 | 9.5 | 51.2 | 2.1 | 3.0 | 3.4 | Sc25 | .52 | 28.1 | 16.8 |
| *RPE65* | 5.8 | 16.2 | C-18 | C-8 | UN | .52 | Sc107 | Sc269 | 20.2 | 27.8 |

**Linkage group name:** *RFLB-RLN2/RLN1/insl4/insl6*

**Location in human genome**: Chromosome IX

| **Gene name** | **Map position in teleosts**  **(Chromosome numbers given in roman numerals, map positions presented with arabic numerals )** | | | | | | | | | |
| --- | --- | --- | --- | --- | --- | --- | --- | --- | --- | --- |
|  | *G. aculeatus* | | *D. rerio* | | *T. nigroviridis* | | *T. rubripes* | | *O. latipes* | |
|  | XIV | XIII | V | XXI | IV | XII | 84 | 221 | XII | IX |
| *DMRT1* |  | 19.2 | 35.3 |  |  | 4.0 |  | 1.9 | Un |  |
| *SMARCA2* |  | 19.1 | 35.07 |  |  | 3.9 |  | 1.8 | Sc_407 |  |
| *KIAA0020* | 10.2 |  | C_10 |  |  | ? | Sc216  ? |  | 28.5 |  |
| *RFX3* | 6.06 |  | C_10 |  | 2.1 |  | .71 |  | 12.5 |  |
| *CBC37L1* | 7.9 |  | C_10 | 7.9 | .83 |  | Sc213  ? |  | 15.4 |  |
| *JAK2* | 7.2 | 17.2 | 59.34 | 44.2 | 1.97 | UN | Sc243  ? | Sc243  ? | 4.8 | UN |
| ***insl6*** | -- | -- | -- | -- | -- | -- | -- | -- | -- | -- |
| ***insl4*** | -- | -- | -- | -- | -- | -- | -- | -- | -- | -- |
| ***RLN2*** | 7.2 |  | -- | -- | 10.8 |  | .09 |  | 14.8 |  |
| ***RLN1*** | -- | -- | -- | -- | -- | -- | -- | -- | -- | -- |
| *C9orf46* | 7.4 |  | -- |  | 1.0 |  | .09 |  | 14.7 |  |
| *K1AA1432* | 5.89 |  |  | 6.8 | 2.23 |  | .82 |  | 12.3 |  |
| *ERMP1* | 5.92 |  |  | 46.0 | 2.22 |  | .80 |  | 12.4 |  |
| *MLANA* | 5.92 |  | A |  | 2.19 |  | .77 |  | 12.4 |  |
| *GLDC* | 3.98 |  | 59.6 |  |  | 5.21 |  | 3.2 |  | 3.9 |
| *UHRF2* | 5.99 |  | UN |  | 2.2 |  | .78 |  | 12.4 |  |

**Linkage group name:** *RFLCI-RLN3*

**Location in human genome**: Chromosome XIX

| **Gene**  **name** | **Map position in teleosts**  **(Chromosome numbers given in roman numerals, map positions presented with arabic numerals )** | | | | | | | | | |
| --- | --- | --- | --- | --- | --- | --- | --- | --- | --- | --- |
|  | *G. aculeatus* | | *D. rerio* | | *T. nigroviridis* | | *T. rubripes* | | *O. latipes* | |
|  | IX | XI | I | III | XVIII | III | 189 | 141 | I | VIII |
| *SMARCA4* | .91 | 2.2 |  | 16.2 | 3.6 |  | Sc207 | Sc65 | 14.2 | 21.1 |
| *CNN1* |  | 2.9 | 47.9 | 62.9 | un | 1.8 | Sc151 |  |  | A |
| *TNPO2* | 15.0 | 11.6 | 50.1 | 15.8 | 2.03 | 8.5 | .345 | .68 | 31.3 | 13.6 |
| *MAST1* | .87 | 3.3 | 48.6 |  | 3.6 |  | Sc207 | Sc139  ? |  | 21.4 |
| *NP11566.1* | 15.0 |  | 51.7 |  | 2.07 |  | Sc189  .389 | ?same | 31.4 |  |
| *C19orf53* | 15.09 |  | 51.5 |  | 2.07 |  | .389 | ?same | 31.4 |  |
| *NANOS3* | 15.1 |  | II |  | 2.08 |  |  |  | 31.4 |  |
| *RFX* | 15.0 | 11.6 |  | 16.1 | 2.04 | 8.54 | .35 | .66 | 31.3 | 13.6 |
| ***RLN3*** | 15.0 | 11.6 | 50.1 | 16.2 | 2.04 |  | .35 | .67 | 3.2 | 13.6 |
| *SAMD1* | 14.5 | 8.2 |  | 9.6 |  | A |  | .67 |  | 9.5 |
| *asf1b* | 14.6 | 8.2 | 52.0 | 9.6 | 2.24 | A | Sc26  ? | .67 | 38.7 | 9.6 |
| *pKN1* | 16.4 | 2.6 | 40.1 |  | 1.3 |  | Sc194  ? | ? | 1.9 | 21.8 |
| *FAM125A* | 14.4 |  | 55.7 |  | Un |  | .57 |  | 39.0 |  |
| *NXNL1* | 14.4 |  | 53.7 |  | 1.5 |  | .38 |  | -- |  |
| *SLC27A1* | 14.4 | 7.9 | 53.2 | 7.07 |  | 11.1 |  | .66 | 39.0 | 9.2 |
| *PGLS* |  | 7.9 | Un | 6.9 |  | 11.1 |  | .67 |  | 9.2 |
| *GLT25D1* | 14.4 | 7.9 | 53.7 |  | 1.5 | A | .37 | .67 | 39.0 | 9.19 |

**Linkage group name:** *RFLCII-insl3*

**Location in human genome**: Chromosome XIX

| **Gene name** | **Map position in teleosts**  **(Chromosome numbers given in roman numerals, map positions presented with arabic numerals )** | | | | | | | | | |
| --- | --- | --- | --- | --- | --- | --- | --- | --- | --- | --- |
|  | *G. aculeatus* | | *D. rerio* | | *T. nigroviridis* | | *T. rubripes* | | *O. latipes* | |
|  | III | VIII | II | VIII | XV | I | 212 | 221 | IV | XVII |
| *MED26* | 8.9 | 9.6 | 20.1 |  | 3.9 | 14.6 | 0.4 | 0.15 | 13.9 | 6.5 |
| *NR2F6* | 8.5 | 8.7 | 19.9 | XI | 3.8 | 14.9 | 0.3 | SC-332 | 15.5 | 14.4 |
| *OCEL1* | 8.5 |  | 19.9 |  | 3.8 |  | 0.31 |  |  | 14.4 |
| *UNC13A* | 7.4 |  | XXII |  | 4.6 | 4 | 25 | 200 | 8.29 | 22.9 |
| *FCH01* |  | 8.51 |  | 17.8 |  | 15.4 |  | 0.24 | 15.8 |  |
| *B3GNT3* |  | 9.7 |  | 10.9 |  | 14.3 |  | .439/.29 | 14.1/11.0 |  |
| ***insl3*** |  |  | 20 |  | 3.8 |  | 0.35 |  |  |  |
| ***JAK3*** |  | 9.8 |  | 9.9 |  | 14.5 |  | 0.25 | 11.0 |  |
| *SLC5A5* |  | 9.8 |  | 9.9 |  | 14.5 |  | 0.26 | 11.0 |  |
| *CCDC124* |  | 9.8 |  | 9.9 |  | 14.5 |  | 0.28 | 11.06 | XIII |
| *KCNN1* | 8.5 | 9.8 | 19.9 | 9.8 | 38.5 |  | 0.32 | 0.29 | 11.07 | 14.49 |
| *ARRDC2* |  | 9.2 | XI |  |  | 14.3 |  | 208 | 14.8 |  |
| *MAST3* | 8.3 | 9.7 | 20.1 | UN |  | 14.3 | 0.42 | Sc221  .241 | 14.1 | 6.4 |
| *PiK3R2* | 8.3 | 9.4 | 20.1 |  | 3.9 | 14.7 | 0.45 | Sc208  .481 | 14.4 |  |
| *RAB3A* | 8.3 | 1.6 | 20.3 | XX | 4.0 |  | 0.5 | upd | 14.6 | 6.3 |
| *PDE4C* | 8.3 | 9.4 | 20.2 | 28 | 4.0 | 14.8 | 0.47 | upd | 14.5 |  |
| *JUND* | 8.3 |  | 0.5 |  | 4.0 | 14.8 |  | upd | 14.7 |  |

Table S2. Species, gene name in this paper and (in brackets) as in Park *et al.*, (2008), Ensembl gene ID or NCBI accession number, gene location, intron length, C-domain length and scaffold number for the six relaxin family genes identified in five teleost species: *Tak*

*ifugu rubripes, Tetraodon nigroviridis, Danio rerio, Oryzias latipes, and Gasterosteus aculeatus, and Homo sapiens. Additionally, the NCBI numbers for the sequences of the other species (Salmo salar, Oncorhynchus mykiss, Pimephales promelas, Xenopus tropicalis,Mus musculus, Rattus norvegicus, and Canis familiaris) sequences included in the analyses are given.*

| **Species** | **Gene** | **NCBI or Ensembl ID** | **Location** | **Intron** | **C length** | **Scaffold** |
| --- | --- | --- | --- | --- | --- | --- |
| *T. rubripes* | *rln3a (RFLC1)* | SINFRUT00000137500 | 355,373-355,903. | 72 | 200 | scaffold 189 |
|  | *rln3b* *(RFLC2)* | SINFRUT00000156614 | 677,851-678,565. | 340 | 197 | scaffold 141 |
|  | *rln* *(RFLB)* | SINFRUT00000178227 | 93,438-93,788. |  |  | scaffold 243 |
|  | *insl5a* *(RFLA1)* | NM_001122866 | 1318524-1318598 |  |  | scaffold 55 |
|  | *insl5b* *(RFLA2)* | EU437462 | 545257-545331 |  |  | scaffold 166 |
|  | *insl3* (*RFLCII*) | SINFRUG00000162280 | 348,305-364,241. |  |  | scaffold 212 |
| *T. nigroviridis* | *rln3a* *(RFLC1)* | GSTENG00026277001 | 18:2,045,252-2,045,785. | 75 | 200 | SCAF14786 |
|  | *rln3b* *(RFLC2)* | Not identified |  |  |  |  |
|  | *rln* *(RFLB)* | GSTENG00008671001 | 4:1,081,257-1,081,342 |  |  | SCAF10492 |
|  | *insl5a* *(RFLA1)* | EU437466 | 1:15,664,564-15,664,647 |  |  | SCAF15039 |
|  | *insl5b* *(RFLA2)* | EU437463 | 15:3,444,213-3,444,287 |  |  | SCAF14367 |
|  | *insl3* (*RFLCII*) | ENSTNIT00000014190 | 15:3,985,602-3,986,336 |  |  | SCAF14667 |
| *D. rerio* | *rln3a(RFLC2)* | ENSDARG00000070780 | 3:15,511,082-15,514,543. | 2739 |  | Contig BX546473.10. |
|  | *rln3b(RFLC1)* | ENSDARG00000039854 | 1:50,171,667-50,173,460 | 1320 | 215 | Chunk Zv6_scaffold3487.6. |
|  | *rln* *(RFLB)* | Not identified |  |  |  |  |
|  | *insl5a* *(RFLA1)* | ENSDARG00000070966 | 6:15,967,670-15,971,708 |  |  | Contig BX088595.7. |
|  | *insl5b* *(RFLA2)* | ENSDARG00000069294 | 2:6,248,300-6,249,555. | 926 |  | Contig CU104710.7_00050. |
|  | *insl3* (*RFLCII*) | ENSDARG00000035862 | 2:20,047,301-20,048,256 | 401 |  | Contig BX530024.6. |
| *O. latipes* | *rln3a* *(RFLC1)* | ENSORLG00000011777 | 1:31,359,716-31,365,371 |  |  | scaffold502_contig98563 |
|  | *rln3b* *(RFLC2)* | ENSORLG00000010278 | 8:13,648,154-13,648,932. | 332 | 200 | scaffold204_contig71183. |
|  | *rln* *(RFLB)* | ENSORLG00000009974 | 12:14,802,571-14,803,151. | 92 | 224 | scaffold439_contig95544. |
|  | *insl5a* *(RFLA1)* | No ID | 4:16566660-16566743 |  |  | scaffold241_contig77064 |
|  | *insl5b(RFLA2)* | No ID | 17:16,797,142-16,797,481 |  |  | scaffold152_contig61158 |
|  | *insl3*(*RFLCII*) | Not identified |  |  |  |  |
| *G. aculeatus* | *rln3a* *(RFLC1)* | ENSGACG00000018985 | IX: 15,053,435-15,053,844 | 103 | 197 | contig_3135. |
|  | *rln3b* *(RFLC2)* | ENSGACG00000012435 | XI: 11,635,992-11,636,806 | 368 | 200 | contig_4416. |
|  | *rln* *(RFLB)* | ENSGACG00000017364 | XIV:7,439,765-7,441,014 | 402 | 358 | contig_4627. |
|  | *insl5a* *(RFLA1)* | No ID | VIII:8,103,881-8,103,957 |  |  | contig_3688 |
|  | *insl5b* *(RFLA2)* | ENSGACG00000016154 | III:9,587,444-9,587,864 | 94 | 109 | contig_5441. |
|  | *insl3*(*RFLCII*) | Not identified |  |  |  |  |
| *H. sapiens* | *RLN1* | ENSG00000107018 | 9:5,324,969-5,329,873. |  |  | AL135786.17.1.156592 |
|  | *RLN2* | ENSG00000107014 | 9:5,289,868-5,294,969. |  |  | AL135786.17.1.156592 |
|  | *RLN3* | ENSG00000171136 | 19:14,000,017-14,002,783. | 2315 | 178 | AC022098.9.1.237931. |
|  | *INSL3* | ENSG00000105639 | 19:17,788,324-17,819,800. |  |  | AC007201.1.1.41006. |
|  | *INSL5* | ENSG00000172410 | 1:67,036,012-67,039,527. | 2801 |  | AL354978.20.1.98450. |
|  | *INSL6* | ENSG00000120210 | 9:5,153,863-5,175,668. | 21048 |  | AL161450.14.1.171146 |

| **Additional sequences** | **Gene name** | **NCBI**  **accession number** | **Additional**  **sequences** | **Gene**  **name** | **NCBI**  **accession**  **Number** |
| --- | --- | --- | --- | --- | --- |
| *S. salar* | *rln3b* | 57126102 | *O. mykiss* | *rln3a* | 90093954 |
|  | *nsl5a* | 84992813 |  | *insl5a* | 90050284 |
| *P. promelas* | *rln3b* | 7372295 | *M. musculus* | *RLN* | 51093875 |
|  | *insl3* | 73652332 |  | *RLN3* | 27502354 |
| *C. familiaris* | *RLN* | 50978851 |  | *INSL3* | 56119091 |
|  | *RLN3* | 73986786 |  | *INSL5* | 6754357 |
|  | *INSL3* | 50950184 |  | *INSL6* | 7305184 |
|  | *INSL6* | XM_533544 | *X. tropicalis* | *Rln3* | EU437449 |
| *R. norvegicus* | *RLN* | 7242202 |  | *Rln* | EU437458 |
|  | *RLN3* | 24250852 |  | *insl3* | EU493152 |
|  | *INSL3* | NM_053680 |  |  |  |
|  | *INSL6* | NM_022583 |  |  |  |
|  |  |  |  |  |  |

Table S3. - Teleost relaxin family genes and characteristic B chains and B/C and C/A dibasic motifs. X represents multiple possible residues, (Hy) represents a small hydrophobic residue such as valine or isoleucine, and + represents positively charged arginine or lysine.

| **Gene** | **B chain motif** | **B/C dibasic motif** | **C/A dibasic motif** |
| --- | --- | --- | --- |
| *rln3a* | YGV+LCGREFIRAVIFTCGGS | RW++ | RKXRD |
| *rln3b* | YGV+LCGREFIRAVIFTCGGS | RW++ | RKXRD |
| *rln* | YGV+LCGREFIRAVIFTCGGS | RW++ | +X+RN |
| *insl5a* | XXVK(L/M)CGREF(Hy)RAVVYTCGGS | RWRR | XX+R |
| *insl5b* | XX(Hy)+LCGRXF(Hy)RA(Hy)V(Hy)XCGGS | RW+R | (R/H)X(R/W)R |
| *insl3* | XX(Hy)KXCGR(D/E)X(Hy)RXXVXSCGXX | RXXR | RXRR |
