# Supplementary material for: Relaxin gene family in teleosts: phylogeny, syntenic mapping, selective constraint, and expression analysis
Source: BMC Evol Biol. 2009 Dec 16;9:293. doi: 10.1186/1471-2148-9-293 (PMC2805637; doi:10.1186/1471-2148-9-293)

Supplemental Figure S1. Phylogenetic tree based on Bayesian inference using amino acid sequence data based on the WAG + Γ model of sequence evolution of the amino acid sequence data for relaxin family genes amongst teleost species. Confidence in nodes is the Bayesian Posterior Probability based on all sampled trees minus those excluded as burnin. Genes located at each of the four relaxin family loci, *insl5 (RFLA), rln (RFLB), rln3 (RFLCI)* and *insl3 (RFLCII),* are shown in the same colour. Paralogous copies of *insl5* (*insl5a* and *insl5b*) and *rln3* (*rln3a* and *rln3b*) that arose after the teleost WGD are indicated. Mammalian *INSL6* is a tandemly duplicated member of the relaxin family that is linked and paralogous to mammalian *RLN*.


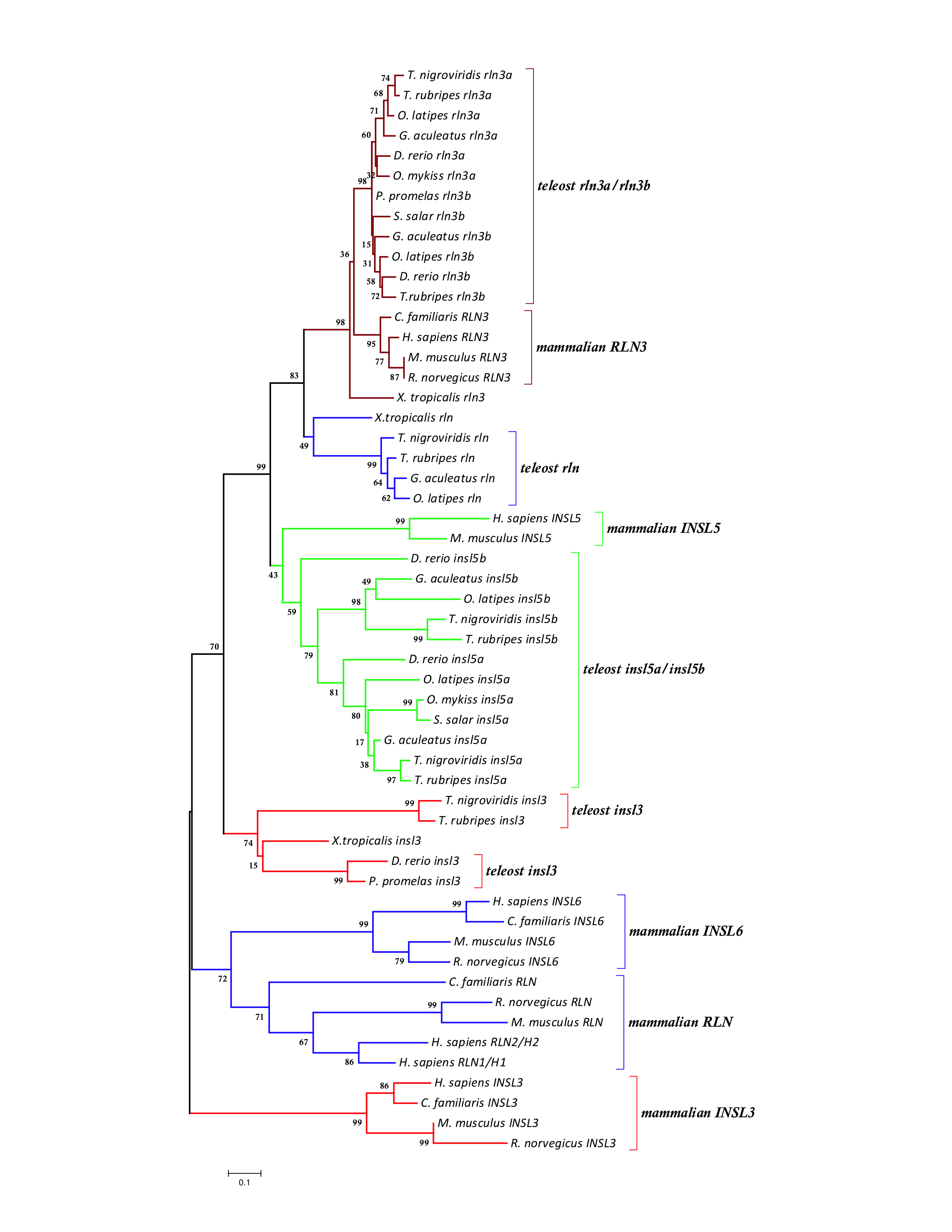

Supplement: Additional file 2 — Bayesian phylogenetic tree based on relaxin family protein sequences. The figure legend and Bayesian phylogenetic tree (in colour) based on amino acid sequences of relaxin family sequences from teleosts and mammals in pdf format. [file 1471-2148-9-293-S2.DOC]
